# The Safety and Effectiveness of Telemedicine for Cancer-Related Colostomy Care in the Early Stage of Discharge: A Prospective, Randomized, Single-Center Study

**DOI:** 10.1089/tmr.2024.0026

**Published:** 2024-07-23

**Authors:** Haizhou Quan, Hongqiang Wang, Yu’e Yang, Hongwei Yu

**Affiliations:** ^1^ Division of General Surgery, Zhoushan Hospital of Wenzhou Medical University, Zhoushan City, China.; ^2^ Outpatient Department, The First Affiliated Hospital of Haerbin Medical University, Harbin, China.

**Keywords:** telemedicine, cancer-related colostomy, quality of life, complications, self-care ability

## Abstract

**Background:**

There has been an exponential growth in the use of telemedicine services to provide clinical care. However, the safety and effectiveness of telemedicine in cancer-related colostomy care during the early stages of discharge remain unclear. This study aimed to support that the safety and effectiveness of telemedicine in cancer-related colostomy care were not inferior to those of outpatient care.

**Methods:**

This was a prospective randomized noninferiority study. A total of 76 consecutive patients who underwent cancer-related colostomy stoma were enrolled and randomly divided into a telemedicine group or an outpatient group with an equal allocation ratio (1:1). The outpatient group was provided in-person interview mode colostomy care, whereas the telemedicine group was provided video interview mode colostomy care. The stoma-related complications, self-care ability, and quality of life reflected the safety and effectiveness of colostomy care in the early stages of discharge.

**Results:**

The incidence of stoma-related complications within two weeks and one month after discharge was not significantly different between the two groups (*p*_2-weeks_ = 0.772 and *p*_1-month_ = 0.760). The mean NCI-CTCAE score for stoma-related complications was less than level 2. The ESCA and C-COH-QOL-OQ scores were not significantly different between the telemedicine and outpatient groups at two weeks and one month after discharge (all *p* > 0.05).

**Conclusion:**

The results revealed that the safety and effectiveness of telemedicine for cancer-related colostomies in the early stages of discharge were not inferior to those of outpatient care alone.

## Introduction

In China, approximately 100,000 new patients undergo colostomies annually. Rectal cancer is one of the most common causes of colostomy.^1^ Approximately half of patients with rectal cancer undergo colostomy. Furthermore, approximately 20–25% of patients operated with a primary anastomosis end up with a stoma because of anastomotic leakage or unsatisfactory anorectal function.^2^ Although surgery could improve the survival rate of patients with rectal cancer, colostomy changes the original method of defecation and with no control defecation at will, which can inevitably cause problems and affect their quality of life.^3^ Most patients undergoing colostomy were excessively dependent on medical staff, did not know much about colostomy care and the prevention of complications, and were maladjusted to colostomy. Patients undergoing colostomy have a high demand for colostomy care during the early stages of discharge. If colostomy care is improper, complications can easily observe.^4^ Effective stoma care can significantly improve patient outcomes and decrease hospital readmission and emergency surgery rates.^5^ The development of new surgical techniques has decreased the length of hospital stay. Currently, most patients undergoing colostomy are discharged from the hospital approximately one week after the operation.^6^ Patients who undergo colostomy mainly adopt a home-based colostomy nursing rehabilitation mode after discharge. Therefore, health care guidance from professionals should improve the quality of colostomy care.^7^ Conventional outpatient care was the primary route for receiving health guidance before the COVID-19 pandemic.

Unfortunately, COVID-19 has spread worldwide since 2019, resulting in colostomy care postponing routine outpatient care and passively shifting services to telemedicine.^8^ The increased availability of telemedicine services has undoubtedly reduced disease exposure among staff members and patients. When using telemedicine management, communication between medical staff and patients is broken through time and place restrictions. Telemedicine can provide rapid access for patients who are not immediately available for colostomy. Some studies have reported high levels of satisfaction with telehealth appointments for gastroenterology,^9^ psychiatric conditions,^10^ diabetes,^11^ and liver diseases.^12^ There has been exponential growth in the use of telemedicine services to provide clinical care currently.^13^ The United States 2021 National Health Interview Survey showed that 35.3% of adults have had telemedicine visits with a health care professional in the past 12 months.^14^ Approximately 1800 patients used telemedicine services at our health center between January 2021 and January 2022. Practical evidence showed that virtual visits would not only substitute for routine health checks but would also substitute for in-person care.

Although this evidence highlights the potential of telemedicine, its use varies across specialties. The heterogeneity of the disease requires further validation before telemedicine can be generalized. However, there is limited practical evidence regarding the impact of telemedicine on patients undergoing colostomy. The safety and effectiveness of telemedicine in cancer-related colostomy care during the early stages of discharge remain unclear. Thus, the safety and efficacy of telemedicine for cancer-related colostomy care in the early stages of discharge have gradually become a major point of interest. This study aimed to demonstrate that the safety and effectiveness of telemedicine for cancer-related colostomy care in the early stages of discharge were not inferior to those of outpatient care.

## Methods

### Study design

This was a prospective, randomized, noninferiority, single-center study. The study was conducted in accordance with the Declaration of Helsinki and approved by the hospital’s Institutional Review Board (Clinical Research Ethics Committee of Zhoushan Hospital). This study aimed to support the statement that the safety and effectiveness of telemedicine for cancer-related colostomy care in the early stages of discharge are not inferior to those of outpatient care. The required sample size was determined using a noninferiority test. Considering the possibility of drop outs, the sample size for each group was set to 38. A total of 76 consecutive patients with cancer who underwent permanent colostomy stoma were enrolled and randomly divided into telemedicine and outpatient groups with an equal allocation ratio (1:1). The telemedicine group was given the video interview mode of colostomy care at two weeks and one month after discharge, whereas the outpatient group was given in-person interviews with colostomy care. The incidence and severity of stoma-related complications reflected the safety of colostomy care, whereas the use of self-care abilities and quality of life reflected the effectiveness of colostomy care in this study. During the early stages of discharge, data on stoma-related complications, self-care ability, and quality of life were collected and compared.

### Patient selection

The inclusion criteria were as follows: (1) patients willing to conduct follow-up observation and have signed the informed consent; (2) patients aged 18–70 years, regardless of sex; (3) patients with confirmed rectal adenocarcinoma, irrespective of tumor stage; (4) patients who underwent permanent colostomy stoma within 10 days and were in accordance with the criteria for discharge with a new ostomy from home health care; and (5) the life expectancy of patients after surgery was >6 months.

The exclusion criteria were as follows: (1) patients who were currently receiving therapy or case management from a mental health worker; (2) patients with serious heart and lung diseases that restricted their participation in the study; (3) patients who had poor compliance with mental disorders; and (4) patients with disturbances in communication, reading, or comprehension.

### Interventions

All patients had follow-ups at two weeks and one month after discharge. At every follow-up, the stoma therapist and fistula nurses viewed the patient’s stoma, pouching system, and peristomal skin; evaluated the quality of life and self-care ability of the patient; documented any stoma-related complications; and educated them on colostomy care, mental care, and complication management and prevention (approximately two hours). Colostomy care refers to the clinical practice guidelines for the nursing care of adult patients with stomas. The outpatient group was provided in-person interview mode colostomy care, whereas the telemedicine group was provided video interview mode colostomy care. In the telemedicine group, video interviews were conducted between the stoma therapist and fistula nurses using the hospital’s remote medical platform, and with patients using smartphones. Meanwhile, a WeChat applet called “virtual colostomy care clinic” built by a network engineer can provide physician–patient interactions.

### Observation index

(1) The incidence and severity of stoma-related complications were assessed to evaluate the safety of colostomy care. Common early ostomy adverse events include stomal bleeding, stomal necrosis, skin and mucosal separation, stomal retraction, stomal prolapse, stomal stenosis, and peristomal dermatitis. The NCI Common Terminology Criteria for Adverse Events (NCI-CTCAE) was used to evaluate the severity of complications. The NCI-CTCAE was classified into levels 1–5 based on severity. Higher levels indicate more serious complications.
(2) The Exercise of Self-care Agency Scale (ESCA) was used to evaluate patients’ self-care abilities. The ESCA was developed by Kearney in 1979 and focuses on individuals’ self-assessment of their interest in self-care activities.^15^ The ESCA scale includes four dimensions: self-care knowledge, self-care responsibility, self-care skills, and self-concept, with an aggregate score of 172. Higher scores indicate stronger self-care abilities.
(3) The Chinese version of the City of Hope scale was used to evaluate QOL. The City of Hope-Quality of Life-Ostomy Questionnaire (COH-QOL-OQ) was originally developed through a grant to assess QOL in persons living with an ostomy.^16^ The Chinese version of C-COH-QOL-OQ was developed by Gao and colleagues.^17^ The C-COH-QOL-OQ includes four dimensions: physical, psychological, social, and mental health. The domain scores ranged from 0 to 10, with higher scores indicating a better quality of life.

### Data collection and statistical analysis

All continuous variables are expressed as median values, and categorical variables are expressed as numbers and percentages. Comparisons of continuous variables between groups were performed using the Student’s *t-test*. Categorical variables were compared using the χ^2^ test or Fisher’s exact test as appropriate.

Statistical significance was set at *p* < 0.05. SPSS Statistics (version 20.0; IBM Corp., Armonk, NY, USA) was used for all the analyses.

## Results

### Background characteristics

A total of 76 consecutive patients who underwent cancer-related colostomy between November 1, 2022, and February 28, 2023, were enrolled and randomly divided into a telemedicine group or an outpatient group with an equal allocation ratio (1:1). Thirty-eight patients who received telemedicine care dropped out of the study because of acute mechanical intestinal obstruction within two weeks of discharge. A total of 38 patients who underwent outpatient care dropped out of the study because of failure to follow up as required. The detailed background characteristics of the patients in the outpatient and telemedicine groups are summarized in Table 1. No significant differences were observed in background characteristics between the two groups (*p* > 0.05) (Table 1).

**Table 1.** Baseline Demographic of the Study Sample by Groups

Demographics	Outpatient group (*n* = 37)	Telemedicine group (*n* = 37)	*p* values
Age; (mean ± SD)	61.71 ± 10.26	63.56 ± 9.66	*p* = 0.084
Gender; *n* (%)			*p* = 0.619
Male	24 (64.9%)	26 (70.3%)	
Female	13 (35.1%)	11 (29.7%)	
Marital status; *n* (%)			*p* = 0.556
Single	2 (5.4%)	1 (2.7%)	
Married	35 (94.6%)	36 (97.3%)	
Living status; *n* (%)			*p* = 0.496
Living alone	4 (10.8%)	6 (16.2%)	
Living with family	33 (89.2%)	31 (83.8%)	
Education; *n* (%)			*p* = 0.710
Elementary school	19 (51.4%)	22 (59.5%)	
High school	16 (43.2%)	14 (37.8%)	
College/university	2 (5.4%)	1 (2.7%)	
Residence; *n* (%)			*p* = 0.480
City	23 (62.2%)	20 (54.1%)	
Rural areas	14 (37.8%)	17 (45.9%)	
Income; *n* (%)			*p* = 0.286
≤25,000 (CNY/y)	31 (83.8%)	34 (91.9%)	
>25,000 (CNY/y)	6 (16.2%)	3 (8.1%)	
Neoadjuvant treatment; *n* (%)	30 (81.1%)	29 (78.4%)	*p* = 0.772
Postoperative chemotherapy; *n* (%)	31 (83.8%)	32 (86.5%)	*p* = 0.761
Surgical approach; *n* (%)			*p* = 0.744
Laparotomy	6 (16.2%)	5 (13.5%)	
Laparoscopy	31 (83.8%)	32 (86.5%)	

SD, standard deviation.

### Safety of telemedicine for colostomy care

Within two weeks after discharge, the stoma-related complications were peristomal irritant dermatitis (4, 10.8%), separation of the skin and mucosa (1, 2.7%), stomal retraction (1, 2.7%), stomal stenosis (1, 2.7%), and stomal ischemia (1, 2.7%) in the outpatient group. In the telemedicine group, complications included peristomal irritant dermatitis (4, 10.8%), separation of the skin and mucosa (1, 2.7%), stomal retraction (1, 2.7%), and stomal ischemia (1, 2.7%). The incidence of stoma-related complications between the groups showed insignificant differences within two weeks after discharge (21.6% vs. 18.9%; *p* = 0.772), as illustrated in Table 2. The NCI-CTCAE score for stoma-related complications was less than level 2 within two weeks of discharge. Patients with complications did not require hospitalization.

**Table 2.** Comparison of Ostomy-Related Complications Between Two Groups

Complications; *n* (%)	Two weeks after discharge		One month after discharge	
	Outpatient group	Telemedicine group	Outpatient group	Telemedicine group
Peristomal irritant dermatitis				
erythema	1 (2.7%)	1 (2.7%)	1 (2.7%)	1 (2.7%)
papules	1 (2.7%)	1 (2.7%)	1 (2.7%)	0
skin erosions	1 (2.7%)	1 (2.7%)	1 (2.7%)	1 (2.7%)
ulcers	0	0	0	0
vesicles	1 (2.7%)	1 (2.7%)	0	1 (2.7%)
Stomal bleeding	0	0	0	0
Stomal necrosi	0	0	0	0
Skin and mucosal separation	1 (2.7%)	1 (2.7%)	1 (2.7%)	1 (2.7%)
Stomal retraction	1 (2.7%)	1 (2.7%)	2 (5.4%)	2 (5.4%)
Stomal prolapse	0	0	0	0
Stomal stenosis	1 (2.7%)	0	0	0
Stomal ischemia	1 (2.7%)	1 (2.7%)	0	0
Stomal hernia	0	0	0	1 (2.7%)
Total; *n* (%)	8 (21.6 %)	7 (18.9%)	6 (16.2%)	7 (18.9%)
*p* values	*p* = 0.772		*p* = 0.760	

Within one month after discharge, the stoma-related complications were peristomal irritant dermatitis (3, 8.1%), separation of the skin and mucosa (1, 2.7%) and stomal retraction (2, 5.4%) in the outpatient group, whereas in the telemedicine group, the complications were peristomal irritant dermatitis (3, 8.1%), separation of the skin and mucosa (1, 2.7%), stomal retraction (2, 5.4%), and stomal hernia (1, 2.7%). The incidence of stoma-related complications between the groups showed insignificant differences within one month of discharge (16.2% vs. 18.9%; *p* = 0.760), as illustrated in Table 2. The NCI-CTCAE score for stoma-related complications was less than level 2 within one month of discharge. Patients with complications do not need to be hospitalized.

### Effectiveness of telemedicine for colostomy care

The ESCA scores were 101.4 ± 13.9 and 106.3 ± 16.5, respectively, in the telemedicine group, and 101.8 ± 13.5 and 103.3 ± 13.4, respectively, in the outpatient group at two weeks and one month after discharge. The C-COH-QOL-OQ scores were 6.15 ± 0.91 and 6.34 ± 0.86, respectively, in the telemedicine group, and 6.27 ± 0.71 and 6.38 ± 0.61, respectively, in the outpatient group at one weeks and one month after discharge. The ESCA and C-COH-QOL-OQ scores were not significantly different between the telemedicine and outpatient groups (all *p* > 0.05), as shown in Table 3.

**Table 3.** Comparison of Effectiveness Between Two Groups

Subgroup	Outpatient group	Telemedicine group	*p* values
ESCA scale (mean ± SD)			
Two weeks after discharge	101.8 ± 13.5	101.4 ± 13.9	P = 0.897
One month after discharge	103.3 ± 13.4	106.3 ± 16.5	P = 0.399
C-COH-QOL-OQ scale (mean ± SD)			
Two weeks after discharge	6.27 ± 0.71	6.15 ± 0.91	P = 0.543
One month after discharge	6.38 ± 0.61	6.34 ± 0.86	P = 0.840

SD, standard deviation; ESCA, exercise of self-care agency scale; C-COH-QOL-OQ, Chinese version of City of Hope-Quality of Life-Ostomy Questionnaire (average score).

## Discussion

This study showed that the safety and effectiveness of telemedicine in cancer-related colostomy care were not inferior to those of outpatient care during the early stages of discharge. During the early stages of discharge, some patients with ostomies experienced ostomy-related complications. Hospital readmissions and emergency visits are often associated with ostomy-related complications. Thus, the safety of colostomy management is reflected in the stoma-related complications. This study showed that the incidence of stoma-related complications in the telemedicine and outpatient groups was not significantly different in the early stages of discharge. The NCI-CTCAE scores for stoma-related complications were all less than level 2. Patients with complications did not require hospitalization. Individuals living with ostomies bear the burden of self-care after discharge. The quality of self-care is closely related to the quality of life of people undergoing an ostomy. Currently, the ESCA is considered an effective method for measuring and evaluating self-care ability in adults, and the C-COH-QOL-OQ scales is considered an effective method for measuring and evaluating the quality of life related to population health in China. Thus, the use of the ESCA and C-COH-QOL-OQ scales reflects the effectiveness of colostomy care in this study. The research showed that the ESCA and C-COH-QOL-OQ scores were not significantly different between the telemedicine and outpatient groups in the early stages of discharge. Implementation of telemedicine is feasible for patients receiving cancer-related colostomy care in the early stages of discharge.

In general, telemedicine visits may be better suited for patients with stable chronic conditions. Although the early stages of discharge for patients undergoing colostomy have a high demand for colostomy care, ideally, patients should be able to manage all aspects of ostomy care independently after discharge. Thus, telemedicine is suitable for managing colostomy care. This is one of the main reasons why the safety and effectiveness of telemedicine for colostomy care are not inferior to those of outpatient care in the early stages of discharge. In addition, advances in remote medical technology can provide patients with convenient virtual visits and connect them to care. Telemedicine is the use of real-time two-way telecommunications technologies to provide clinical health care.^18^ Advances in remote medical technology are a prerequisite for the convenience of telemedicine. Lv et al. showed a significant number of mobile phones and high network coverage among the Chinese national population.^19^ Hospitals are universally equipped with remote medical platforms, and smartphones are widely used. Advances in remote medical technology have enabled patients to undergo professional colostomies at home. To further increase the accessibility to telemedicine, public services have constantly strengthened telemedicine equipment, waived the need for telemedicine, and encouraged health care professionals to care for patients using telemedicine.

It should be mentioned that there are advantages to using telemedicine for colostomy care. When using telemedicine management, communication between medical staff and patients breaks through time and place restrictions. Telemedicine can provide rapid access to patients who are not immediately available in person or who live in remote locations. If the safety and effectiveness of telemedicine for colostomy care are not inferior to those of outpatient care, these advantages increase the level of satisfaction with telemedicine.

The present study had some limitations. Telemedicine for cancer-related colostomy care has several benefits for patients, clinicians, and the health care system, but its use faces challenges. For example, the feasibility of telemedicine for colostomy care depends strongly on the internetwork proficiency of clinicians and patients. Some patients have limited access to internet-based services because of insufficient internet skills and acceptance of technology.^20,21^ These challenges can lead to digital inequalities. Therefore, digital inequalities should be considered and overcome when encouraging telemedicine applications. As the telemedicine mode transformed from an aspirational goal to a *de facto* standard, digital inequality for patients in the telemedicine mode should be studied.

## Conclusion

The results of this study revealed that the safety and effectiveness of telemedicine for cancer-related colostomy care in the early stages of discharge were not inferior to those for outpatient care.

## Data Availability

Data included in article/supp. material/referenced in article.

## Abbreviations Used

COH-QOL-OQ: The City of Hope-Quality of Life-Ostomy Questionnaire
C-COH-QOL-OQ: The Chinese version of City of Hope-Quality of Life-Ostomy Questionnaire
ESCA: The Exercise of Self-care Agency Scale
NCI-CTCAE: The NCI Common Terminology Criteria for Adverse Events
